# Validation of genotype imputation in Southeast Asian populations and the effect of single nucleotide polymorphism annotation on imputation outcome

**DOI:** 10.1186/s12881-018-0534-8

**Published:** 2018-02-13

**Authors:** Worachart Lert-itthiporn, Bhoom Suktitipat, Harald Grove, Anavaj Sakuntabhai, Prida Malasit, Nattaya Tangthawornchaikul, Fumihiko Matsuda, Prapat Suriyaphol

**Affiliations:** 1 Molecular Medicine Graduate Program, Faculty of Science, Mahidol University, Bangkok, Thailand; 2 Division of Bioinformatics and Data Management for Research, Department of Research and Development, Faculty of Medicine, Siriraj Hospital, Mahidol University, Bangkok, Thailand; 3 Integrative Computational BioScience Center, Department of Biochemistry, Faculty of Medicine, Siriraj Hospital, Mahidol University, Bangkok, Thailand; 4 Center of Excellence in Bioinformatics and Clinical Data Management, Faculty of Medicine Siriraj Hospital, Mahidol University, Bangkok, Thailand; 5 Unité de Génétique Fonctionnelle des Maladies Infectieuses, Department Genome and Genetics, Institut Pasteur, Paris, France; 6 Centre National de la Recherche Scientifique, URA3012, Paris, France; 7 Systems Biology of Diseases Research Unit, Faculty of Science, Mahidol University, Bangkok, Thailand; 8 Medical Biotechnology Research Unit, National Center for Genetic Engineering and Biotechnology, National Science and Technology Development Agency, Bangkok, Thailand; 9 Division of Dengue Hemorrhagic Fever Research, Department of Research and Development, Faculty of Medicine, Siriraj Hospital, Mahidol University, Bangkok, Thailand; 10 Center for Genomic Medicine, Graduate School of Medicine, Kyoto University, Kyoto, Japan

**Keywords:** Imputation, Reference, Genotype, Pan-Asian SNP, SNP annotation

## Abstract

**Background:**

Imputation involves the inference of untyped single nucleotide polymorphisms (SNPs) in genome-wide association studies. The haplotypic reference of choice for imputation in Southeast Asian populations is unclear. Moreover, the influence of SNP annotation on imputation results has not been examined.

**Methods:**

This study was divided into two parts. In the first part, we applied imputation to genotyped SNPs from Southeast Asian populations from the Pan-Asian SNP database. Five percent of the total SNPs were removed. The remaining SNPs were applied to imputation with IMPUTE2. The imputed outcomes were verified with the removed SNPs. We compared imputation references from Chinese and Japanese haplotypes from the HapMap phase II (HMII) and the complete set of haplotypes from the 1000 Genomes Project (1000G). The second part was imputation accuracy and yield in Thai patient dataset. Half of the autosomal SNPs was removed to create *Set 1*. Another dataset, *Set 2*, was then created where we switched which half of the SNPs were removed. Both *Set 1* and *Set 2* were imputed with HMII to create a complete imputed SNPs dataset. The dataset was used to validate association testing, SNPs annotation and imputation outcome.

**Results:**

The accuracy was highest for all populations when using the HMII reference, but at the cost of a lower yield. Thai genotypes showed the highest accuracy over other populations in both HMII and 1000G panels, although accuracy and yield varied across chromosomes. Imputation was tested in a clinical dataset to compare accuracy in gene-related regions, and coding regions were found to have a higher accuracy and yield.

**Conclusions:**

This work provides the first evidence of imputation reference selection for Southeast Asian studies and highlights the effects of SNP locations respective to genes on imputation outcome. Researchers will need to consider the trade-off between accuracy and yield in future imputation studies.

**Electronic supplementary material:**

The online version of this article (10.1186/s12881-018-0534-8) contains supplementary material, which is available to authorized users.

## Background

Genome-wide association studies (GWAS) have been widely used as a reliable method for identifying genetic variants associated with a trait or complex disease. A high density of SNPs increases the chance of finding either a causal mutation for the trait or SNPs close enough to the mutation to confidently suggest a gene or another sequence feature underlying the trait. One way to overcome this problem is using imputation, a process in which samples are genotyped using a low-density SNP array and imputed with information from a reference panel genotyped on a high-density SNP array. This method will also recover genotypes that are missing because of technical issues.

Imputation has successfully helped to identify genetic susceptibilities to various diseases and phenotypes that were not recognized in a genotyped panel [1, 2]. The method relies on the number of SNPs being shared between the two panels and the amount of linkage disequilibrium (LD) between genotyped and non-genotyped SNPs [3]. A low average LD will reduce the accuracy and might require more typed SNPs. The quality of imputation also depends on the choice of reference [1]. If the reference contains genetic variants not present in the actual sample population, it will increase the noise in the data and reduce the usefulness of the imputation. One study of malaria resistance in Gambian children only identified a previously known hemoglobin S variant in the hemoglobin-β gene when a Gambian-specific reference was used [1]. Although this problem is more likely to occur in Africa, where there is a considerably lower LD compared to Europe and Asia [4], determining how to choose the best reference is relevant for any study performing imputation with publicly available reference sets.

Many studies have validated the accuracy and reliability of imputation [5–7], but most of these studies focused on populations of European descent [5, 7]. One study showed that the accuracy of using a publicly available database varied across human populations with Europeans having the highest accuracy and Africans having the lowest [6]. Because Asian populations have some unique genetic characteristics [8], it is not always possible to directly adapt information about genetics or genomics from studies in Caucasian populations [9].

Several types of software are currently available for performing genotype imputation [10–13]. Similarly, many publicly available genetics databases are accessible for public use [14, 15]. One of these is the Pan-Asian SNP genotyping database (PanSNPdb), which collects SNPs and copy number variations from 1719 samples in 71 populations from China, India, Indonesia, Japan, Malaysia, the Philippines, Singapore, South Korea, Taiwan, and Thailand [16, 17]. The genotyping process was performed using the Affymetrix GeneChip Human Mapping 50 K Xba Array.

Most of the studies on imputation have looked at the overall outcome of all SNPs [5, 18], and a few have focused on a particular region within a gene, not the whole genome [19, 20]. We proposed two objectives for the current study. The first was to identify the most preferred reference for imputation in Southeast Asian populations. Using two publicly available haplotype databases, the International HapMap Project (HMII) and the 1000 Genomes project (1000G), we compared the accuracy and yield of imputation in several Southeast Asian populations. Additionally, we looked at imputed results using genotyped samples from a study of a Thai genome cohort. The second objective was to evaluate the imputation results of different regions in the human genome using a real dataset from the Thai dengue study as a model. This is the first extensive study of imputation in Southeast Asian populations and the first illustration of imputation differences between SNPs in different regions of the genome.

## Methods

This study was divided into two parts. The first part aimed at showing the difference in imputation accuracy by using different criteria for selecting a reference database. Additionally, using data from populations within the Southeast Asian region illustrated the variation in accuracy when going from one population to another. The second part used real genotype data in all autosomes to classify SNPs into different groups according to their location within genes. Imputation accuracy, GWAS significance and allele frequency were then correlated with the classification.

### Sample datasets

We performed the first part of our analysis using data from PanSNPdb [16]. To illustrate the imputation accuracy in Southeast Asian populations, we selected all available samples from Indonesia (ID, *n* = 288), Malaysia (MY, *n* = 217), the Philippines (PI, *n* = 125), Singapore (SG, *n* = 90), and Thailand (TH, *n* = 245). Only SNPs that were polymorphic in all populations were used in this study (*n* = 52,160).

The second part was imputation accuracy and yield in a patient dataset in which we had access to phenotypes because the phenotypes allowed us to observe the effect of imputation on subsequent association tests. The subjects were 609 Thai dengue patients who were 1–15 years-old from Siriraj, Ramathibodi, and Khon Kaen hospitals. A total of 468,987 SNPs from Illumina Human Hap610 array (Illumina Inc., San Diego, CA) passed the quality control requirements (QC). The accuracy of imputation was tested for each SNP from the dengue dataset by first randomly choosing half of the SNPs from the genotyping panel to create a mutually exclusive set of SNPs: *Set 1* and *Set 2*. Then, *Set 1* SNPs were used to impute *Set 2* to create a complete SNP panel*. Set 2* was also used to impute *Set 1* to create a complete SNP panel. Based on our results in the previous section, HMII data were used as a reference for imputing the SNPs from the dengue dataset. The total number of SNPs after imputation was 1,417,081. Post-imputation QC reduced these numbers to 858,480.

### Quality control and multidimensional scaling

For all of the sample datasets, QC was performed in PLINK v1.07 [12] using standard procedures for GWAS [21]. We included all markers with a call rate > 0.95, a minor allele frequency (MAF) > 0.01, and a Hardy–Weinberg equilibrium (HWE) > 10^− 7^. Samples with call rates < 0.95 were excluded from the analysis along with samples that had first-degree relationship agreement, as evaluated by expected IBD sharing in PLINK v1.07. Multidimensional scaling (MDS) of Southeast Asian populations from PanSNPdb was performed in PLINK v1.07. This method allowed for visualization of principle components in the admixed population. Plotting of the MDS was conducted in R version 3.0.2 (http://www.r-project.org/).

### Imputation procedure

In the first part of the study, each population from PanSNPdb was analyzed independently. Five percent of SNPs were randomly selected and removed. The same SNPs set of the removed SNPs were applied to all populations. SHAPEIT version 2 software was used to pre-phase the SNPs [22]. Imputation was accomplished with IMPUTE2 to recover the removed SNPs [23]. Each population was phased and imputed using both references in turn. According to guidelines from IMPUTE2, we imputed each chromosome separately and used windows of 5 Mb with an additional 250 kb buffer region on both sides of the analysis interval. The options used in the program were -buffer 1000, −iter 30, −burnin 10, and -k 80. The processes for random removal, phasing, and imputation were repeated five times.

The second part of our study used all the autosomal SNPs from the dengue dataset. Half of the autosomal SNPs from Thai dengue patients were removed by every second SNP (*Set 1*). Another dataset (*Set 2*) was then created in which the other half of the SNPs (*Set 1*) were removed. In this way, all SNPs were imputed once. Imputation was performed with HMII as described above. After imputation, SNPs were filtered using a QC process similar to the initial filtering of raw genotypes. Post-imputation QC excluded SNPs with MAF < 0.01, call rate < 0.95, and HWE < 5 × 10^− 7^. These datasets were used in the GWAS analyses. We then selected only the imputed SNPs from the two datasets and merged them into a single dataset in which all SNPs had been imputed. This dataset was used to compare imputation accuracies of SNPs according to their location relative to known genes.

References used for the imputation were downloaded prior to the imputation process from the Impute website (http://mathgen.stats.ox.ac.uk/impute/impute.html). The references were labeled on the website as International HapMap project phase II release #22 (HMII) and 1000G phase I. A total of 1,417,081 SNPs from 90 Chinese and Japanese samples were used from HMII with an additional 39,343,900 SNPs from 1092 worldwide sample populations in the combined reference from 1000G. PanSNPdb shared 47,870 SNPs with the HapMap reference and 51,849 with the 1000G reference. The Thai dengue dataset shared 493,846 SNPs with the HapMap reference and 565,912 with the 1000G reference.

### Imputation yield and accuracy

IMPUTE2 gives each imputed genotype a posterior probability score (info score) between zero and one. A higher threshold cut off for the probability score will usually result in higher accuracy but a lower yield. In this study, the posterior probability threshold was set to 0.9 to gain results with a high confidence of accuracy [24]. Genotypes with posterior probabilities < 0.9 were set to missing. Yield was reported as the percentage of non-missing genotypes within the removed SNPs and accuracy as the percentage of imputed, non-missing genotypes that matched the original genotypes.

### SNP selection and annotation

All SNPs from the dengue dataset were grouped based on their location in genes. Gene annotation was collected from Illumina sample sheets (Illumina Inc., San Diego, CA) and the NCBI database of genetic variation [25]. Targeted gene regions included coding, intergenic, intronic, and untranslated regions (UTR). Some SNPs mapped to more than one location and were marked as being in a complex region. There were 53,277 SNPs that were not in any of these 5 groups and were subsequently discarded from further analysis.

The difference between imputed and genotyped data in the dengue dataset was evaluated by looking at several properties of the SNPs. Differences in accuracy and yield for each gene region were measured by varying the posterior probability threshold from 0.5 to 1.0. MAFs and *p*-values from chi-square tests for each SNP were compared between imputed and genotyped datasets. Coefficients of determination (r^2^) were calculated for each comparison to estimate the concordance of imputed and genotyped SNPs. Pairwise LD, which is measured as r-squared, was calculated within a region of < 1 Mb around each SNP. The calculation was performed in PLINK v1.07 using the option --r2 with --ld-window-r2 0 and --ld-window-kb 1000. Average R-squared values for each region were calculated and plotted in Microsoft Excel. R-squared values were also plotted against distance and averaging over 1 kb bins using R software (http://www.r-project.org/).

## Results

### Genotype imputation of Southeast Asian populations

**Fig. 1:**
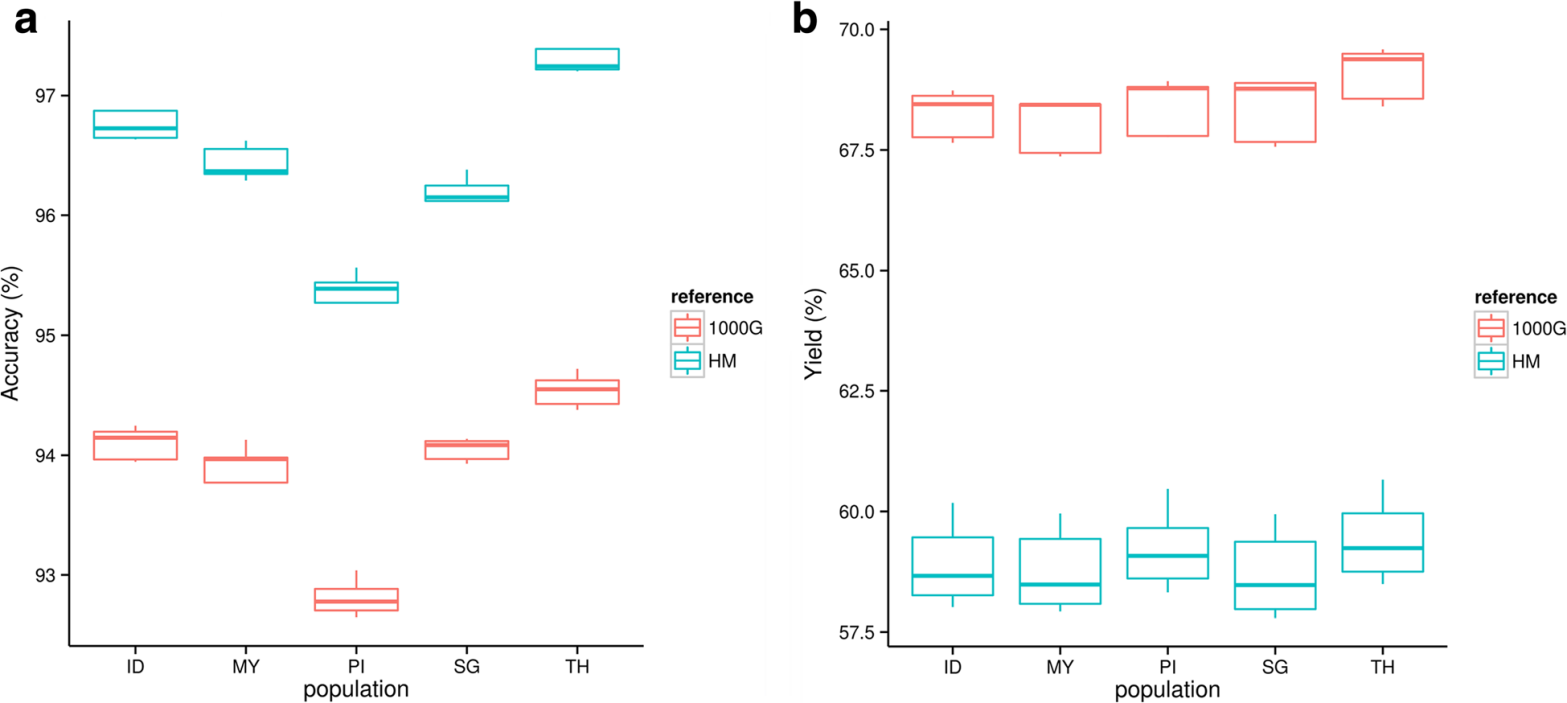
Boxplot of accuracies and yields for imputation results across all populations. Five percent of randomly removed SNPs were imputed with IMPUTE2 using either the 1000 Genomes project phase I (1000G) or combined Chinese and Japanese haplotypes from the International HapMap project phase II (HMII) as a reference. The imputed SNPs were tested for accuracy with the previously removed SNPs. The same set of the removed SNPs was applied to all population dataset. The technique was repeated five times. **a** Boxplot of accuracy comparing populations and references. **b** Boxplot of yield comparing populations and references. Abbreviations: Indonesia (ID), Malaysia (MY), the Philippines (PI), Singapore (SG), and Thailand (TH)

The accuracy and variability of imputation for Southeast Asian samples were assessed with five populations downloaded from PanSNPdb and two publicly available references from the HapMap project and the 1000 genomes project. All populations had an average imputation accuracy of more than 92% (Fig. 1), regardless of which reference was used. Imputation with HMII as a reference gave an average accuracy of 96.57%, while for 1000G, the accuracy was 93.98% (Fig. 1a). The Thai (TH) population had the highest accuracy for both reference panels followed by Indonesia (ID), whereas the population from the Philippines (PI) had the lowest accuracy. The yield for each population was lower when imputation was performed with the HMII reference (average = 59.03%) compared to the 1000G reference (average = 68.44%) (Fig. 1b). The yields for all populations were similar when using the same reference. The only exception was the TH population imputed with the 1000G reference, which had a higher yield compared to the other populations.

**Fig. 2:**
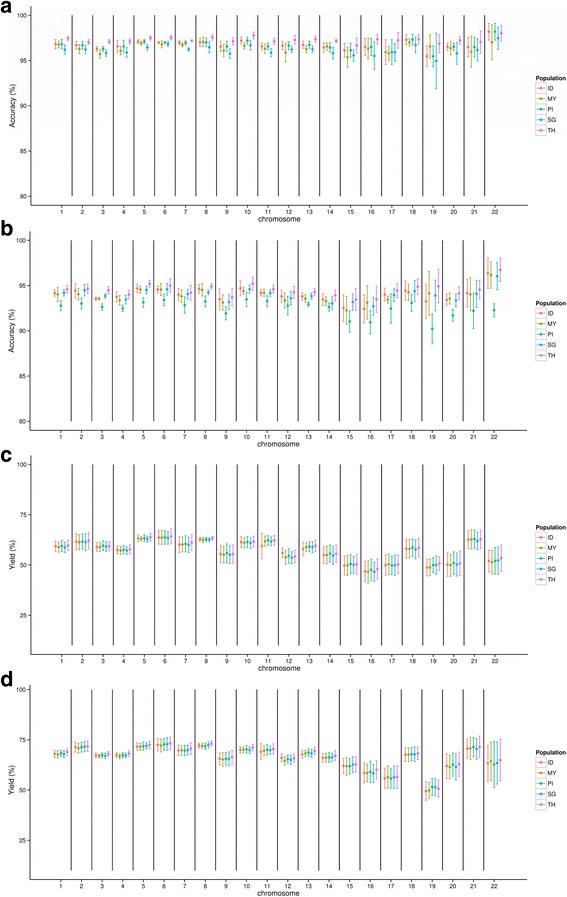
Imputation accuracy and yield by chromosome. The results derived from 5% randomly removed SNPs. Imputation with IMPUTE2 was accomplished to recover the removed SNPs. The imputed SNPs were tested for accuracy with previously removed SNPs. The same set of the removed SNPs was applied to all population dataset. This process was repeated five times. **a** Imputation accuracy by chromosome using HMII as a reference. **b** Imputation accuracy by chromosome using 1000G as a reference. **c** Imputation yield by chromosome using HMII as a reference. **d** Imputation yield by chromosome using 1000G as a reference. Abbreviations: Indonesia (ID), Malaysia (MY), the Philippines (PI), Singapore (SG), and Thailand (TH)

Next, we looked at the results from each chromosome separately to demonstrate the variability of accuracies and yields (Fig. 2). Most results of imputation with HMII as a reference provided more than 95% accuracy (Fig. 2a). The 1000G reference provided a lower accuracy compared to HMII (Fig. 2b). The most striking result was that there was a change in standard deviation for imputation accuracy between the chromosomes. In particular, chromosomes 19 showed the lowest accuracy and higher variation in accuracy. Plotting the yield by population and chromosome showed no significant differences (Fig. 2c, d). However, chromosomes 19 also showed lower yield and chromosome 22 exhibited the highest level of variability. Because the imputation technique is, to a large extent, based on LD, we wanted to see if the higher variance could be correlated to any differences in the LD-pattern. We calculated the LD for each SNP pair with a distance between 10 kb and 1 MB. The number of these pairs that had an LD > 0.2 was recorded for each chromosome (Additional file 1: Figure S1). Chromosome 19 and 22 had the lowest values.

**Fig. 3:**
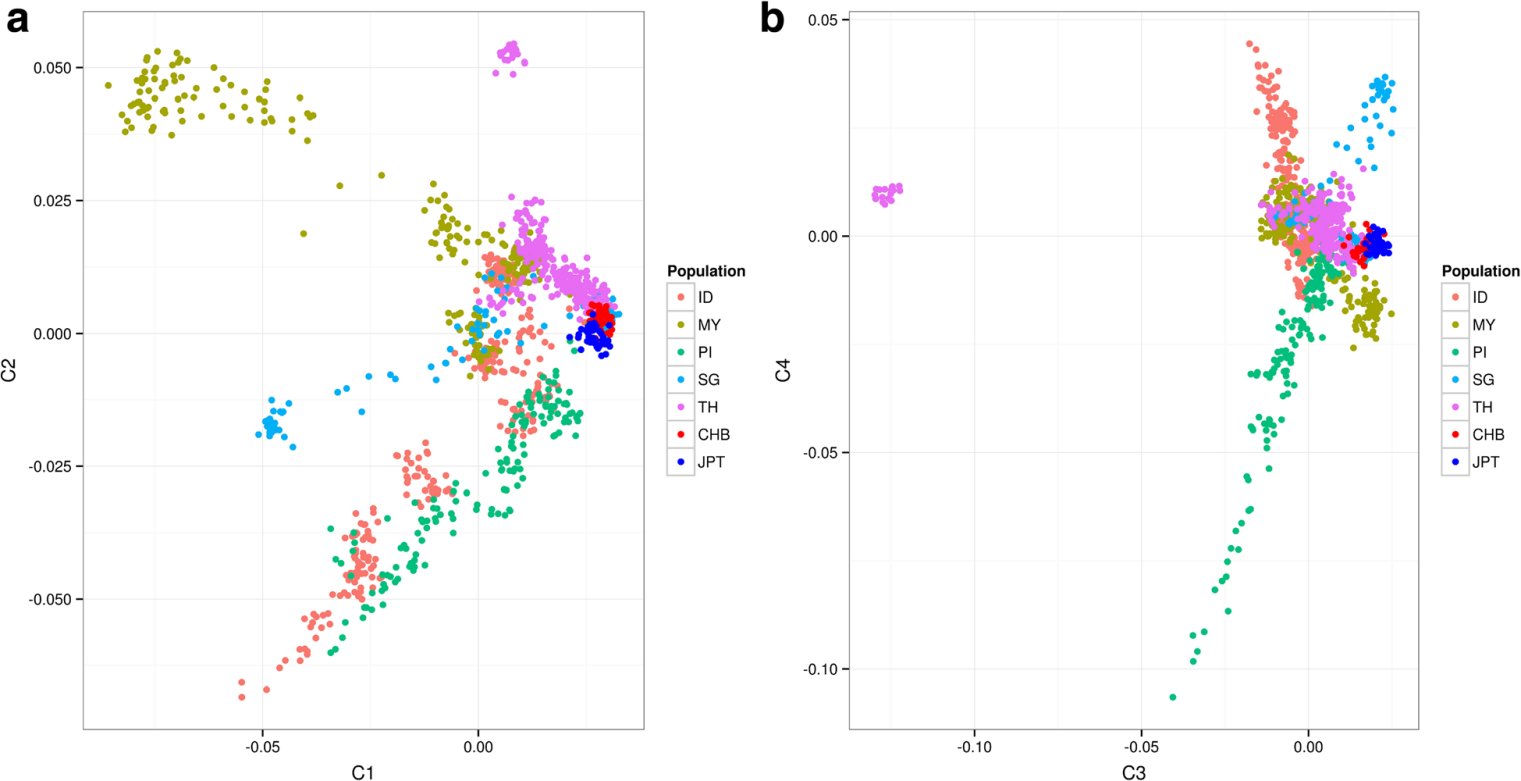
Multidimensional scaling plot of Southeast Asian populations from PanSNPdb. Genotype data of samples from Southeast Asian populations were downloaded from PanSNPdb. After quality control, multidimensional scaling (MDS) was performed in PLINK v1.07. **a** Plotting of the first (C1) and the second (C2) axes. **b** Plotting of the third (C3) and the fourth (C4) axes. Abbreviations: Indonesia (ID), Malaysia (MY), the Philippines (PI), Singapore (SG), Thailand (TH), China (CHB) and Japan (JPT)

Trying to explain the differences in imputation accuracy, we investigated the population diversity of the five populations using MDS (Fig. 3). Whereas most samples were grouped together, all populations except TH showed large internal variation along the primary axis (C1) (Fig. 3a). The secondary axis (C2) mainly described the difference between 18 samples of the Thai Mlabri ethnic group (from Nan Province, Thailand) to the rest of the Thai samples. The third axis (C3) also mainly described the variation within Thai samples, whereas the fourth axis (C4) showed variation within PI, ID and SG (Fig. 3b).

### Effects of SNP location in a gene on imputation

Using the dengue GWAS dataset to evaluate the effects of SNP locations within a gene on the quality of imputation, we found 415,710 SNPs located within genes (Additional file 2: Table S1). As expected, intergenic regions contained the most SNPs, while complex regions, where SNPs have been associated with more than one gene, had the fewest. Varying the threshold settings in IMPUTE2 for accepting an imputed genotype showed that increasing the threshold led to an increase in accuracy but a decrease in yield (Additional file 1: Figure S2). Imputation results for SNPs in coding regions showed the highest yield and accuracy. Intronic and intergenic regions led to the second and third highest yield and accuracy, respectively. At a threshold of 0.5, all regions showed a similar yield. However, with an increasing threshold, the yield of coding regions increased compared to other locations. The opposite effect was observed for accuracy, and all locations approached the same level of accuracy when the threshold approached 1.

We further compared the measured MAF from the initial genotyped data to the MAF of the imputed data (Additional file 1: Figures S3-S4). Then, any imputed SNP that did not pass the quality-criteria (call rate > 0.95, MAF > 0.01, HWE > 10^− 7^) were removed (Additional file 2: Table S1). The MAF of this reduced set of SNPs were similarly compared to the same set of SNPs from the initial genotyped data (Additional file 1: Figure S4). The correlation for each region between the MAF of the genotyped data and both the imputed data and the imputed and post-imputation filtered data were calculated (Table 1). Coding regions had the fewest SNPs failing the QC, while complex regions had the lowest correlation, followed by untranslated regions (UTR), before filtering of the imputed data. After removing low quality SNPs, all regions showed a high correlation in MAF between imputed and actual genotypes. Looking at the direction of change in MAF showed that SNPs with low initial MAF (MAF < 0.25) predominantly (> 80%) had even lower MAFs after imputation. SNPs with an initial MAF close to 0.5 had an equal distribution of SNPs that obtained higher and lower post imputation MAF. Imputation also appeared to systematically reduce the allele frequency of the initially low MAF for most imputed SNPs (Additional file 1: Figure S5). The same analysis was repeated using *p-*values from the binary association test instead of the allele frequency for each SNP (Additional file 1: Figures S6 and S7). All regions had a generally low correlation for the *p-*values before removing low quality SNPs (*r* < 0.4). After post-imputation QC, all regions showed an increase in correlation. Coding regions had the highest correlation both before (r^2^ = 0.39) and after (r^2^ = 0.81) removing low-quality SNPs. The lowest correlation for the binary association was found in complex regions before post-imputation QC (r^2^ = 0.27) and UTR regions after the QC (r^2^ = 0.76).

**Table 1:** Squared correlation of allele frequencies and chi-square *P-*values from SNPs in different regions

Region	Squared correlation (r^2^) of minor allele frequency		Squared correlation (r^2^) *p*-value from chi-square	
	Before post-imputation QC	After post-imputation QC	Before post-imputation QC	After post-imputation QC
Coding region	0.868	0.997	0.387	0.813
Complex region	0.817	0.991	0.267	0.782
Intergenic region	0.864	0.996	0.328	0.789
Intron region	0.863	0.995	0.340	0.784
UTR region	0.830	0.991	0.303	0.756

To test whether coding regions had a stronger LD compared to the other regions, PLINK v1.07 was used to calculate pairwise LD between each SNP and any other SNP within 1 Mb. LD values, presented as r-squared, are shown as averages for each region (Additional file 1: Figure S8) and as LD vs. distance plot (Additional file 1: Figure S9). SNPs within coding regions had the highest average LD to neighboring SNPs (r^2^ = 0.253), followed by SNPs in intronic regions (r^2^ = 0.232).

## Discussion

Imputation of genotyped datasets is a common practice when performing genome-wide association studies. This technique is used to fill in missing genotypes and to increase the density by adding information from SNPs that are not present in the original dataset [5, 26]. Imputation of SNPs that are not available in the dataset serves several purposes. First, if available SNP arrays are designed based on a specific population, such as Europeans, the SNPs may not cover the areas of interest for another population. SNPs important to populations from Southeast Asia might therefore be underrepresented or missing from these arrays. This situation has been reported for populations from Africa [27] and Mexico [28]. Second, if data are collected independently between groups of case and control populations, the datasets might have been genotyped on different SNP sets. Third, GWAS usually requires a high number of SNPs to increase chance to detect association signals. Although current genotyping arrays could contain more than a million markers, imputation still adds more SNPs for denser full genome coverage.

The choice of reference panels can affect the accuracy of imputation through the genetic variation of the samples and the genetic relationship between the samples in the reference panel and the imputed references [6, 27]. We studied these effects in five populations from Southeast Asia. The 1000G reference provided the highest yield, while the HMII reference had the highest accuracy. These results were the same for all Southeast Asian populations. IMPUTE2 software provides the posterior probability score for each imputed genotype. There was increasing accuracy with a decreasing yield when the probability threshold increased (Additional file 1: Figure S2). At the threshold 1.0, the result showed a large drop in yield but only a limited increase in accuracy. At the threshold 0.9, the slope of the yield and accuracy were significantly changed. This threshold might be a good starting point for using IMPUTE2 for Southeast Asian populations.

Genotype imputation of the Thai population had the highest accuracy in the current study. Previous work has shown that in the PanSNPdb database, the Thai population had the highest relationship to the Chinese and Japanese populations out of the other four study populations [16]. The increased accuracy can therefore be explained by this closer relationship. To determine if population diversity influenced the average imputation accuracy, classical MDS was used to display the variation within Southeast Asian populations. The main variation in the MDS plot (C1) was related to the sub-populations in 4 of the 5 main populations, and the only exception was the Thai population (Fig. 3). The Thai samples form a more homogenous group compared to other populations, and this outcome can help explain why they had the most accurate results. Eighteen individuals from the Thai Mlabri group, which is a hunter-gatherer group in Northern Thailand, clustered away from other Thai samples, which is consistent with the findings of previous studies [17, 29]. Nevertheless, further investigation is necessary to understand the effects of population stratification on imputation results.

Plotting the imputation accuracy and yield for each chromosome revealed that the variation within populations was the highest for the smaller chromosomes, especially chromosomes 19 and 22. The same results were also observed for yield. In particular, the population from the Philippines showed a large increase in variability for these chromosomes when using the 1000G reference. This result might indicate a specific issue with the SNP selection for these chromosomes, such as the LD structure being different or the relationship to the reference being lower in these regions. One possible reason for the increased variability was found by looking at the long distance LD in each chromosome. Chromosome 19 and 22 was shown to have the fewest SNPs connected by LD above 0.2 when comparing inter-SNP distances above 10 kb (Additional file 1: Figure S1). The accuracy of resulting imputation for these two chromosomes will therefore be more dependent on which SNPs are removed and will show more variance between replications with randomly selected SNPs. The results also clearly show that only taking the average numbers for accuracy or yield into account will result in overlooking potentially important information.

Coding sequences have been the favored area to search for functional mutations because these sequences are more informative and easier to interpret due to the direct link to a protein and the possibility of functional changes [30–32]. This approach made it useful to compare the imputation results between coding and other SNP regions. Our results show that a higher percentage of SNPs in coding regions passed the post-imputation QC than in other regions. SNPs in coding regions also had the highest accuracy. This outcome correlates well with the results showing coding regions to have the highest LD with the surrounding SNPs (Additional file 1: Figure S8). This is an important factor to consider when discussing significant results because imputed SNPs in coding regions will have a higher accuracy compared to SNPs in less conserved areas. This trend does not mean that we can omit non-coding SNPs because they have been shown to be associated with phenotypes in more than one-third of GWAS [33–35].

Our study also compared GWAS results from a dataset from Thai dengue fever patients to see how imputation affected the reported results. Imputation appeared to systematically reduce the allele frequency of the initially minor allele for most imputed SNPs (Additional file 1: Figure S5). This effect was more pronounced for SNPs with an initial low MAF and will make imputing low frequency alleles difficult. Our results demonstrated that imputation tended to increase the common allele. This outcome was especially problematic if the genotypes are very rare variants [26]. Even if the most significant SNPs had higher significance in the imputed dataset, this trend was not seen when comparing *p*-values from GWAS before and after imputation.

The imputed genotypes were also subjected to QC-filtering, which is similar to the QC being performed on the raw genotype data. It was previously shown that post-imputation QC did not influence the imputation outcome [36]. However, we observed an improvement in the correlation between measured MAF and *p*-values from imputed data and genotyped data. Without the SNPs failing the QC, the correlation between imputed data and genotyped data was close to one for allele frequency and had improved *p*-values. Even if over half the SNPs were removed in this step, the remaining data had higher quality and were more trustworthy. This difference in post-QC improvement could be due to the initial imputation accuracy. Post-imputation QC might be more important if the initial imputation results are less accurate. This result is also supported by a previous experiment that similarly demonstrated Hardy–Weinberg disequilibrium is a crucial step for post-imputation filtering [37].

This study demonstrates that the expected accuracy and yield of imputation in various Southeast Asian populations varies between populations. Our reference comparison of HMII and 1000G in imputation in Thai GWAS showed that using a larger reference provided a higher yield but caused a reduction in accuracy compared to a smaller but more related reference. We also extensively showed the imputation results with respect to SNP localization near genes using Thai genome-wide genotypes as a model. This study provides crucial information for investigators undertaking imputation, especially in Southeast Asian populations.

## Conclusions

This work provides the first evidence of imputation reference selection for Southeast Asian studies and highlights the effects of SNP locations respective to genes on imputation outcome. Researchers will need to consider the trade-off between accuracy and yield in future imputation studies.

## Additional files

Additional file 1: Figure S1. Long distance LD pr. Mb separated by the chromosome for each population LD for each SNP pair with a distance between 10 kb and 1 Mb taken into account. **Figure S2.** Accuracy and yield of imputation between each location of SNPs. **Figure S3.** Comparing minor allele frequencies of SNPs between imputed and actual genotypes before quality control of imputed results separately plot by each SNP location. **Figure S4.** Comparing allele frequencies of SNPs between imputed and actual genotypes after quality control of imputed results separately plotted by each SNP location. **Figure S5.** Proportion of SNPs with lower AF after imputation vs initial AF. **Figure S6.** Comparing *p*-values between cases and controls of SNPs between imputed and actual genotypes before quality control of imputed results separately plotted by each SNP location. **Figure S7.** Comparing *p*-values between cases and controls of SNPs between imputed and actual genotypes after quality control of imputed results separately plotted according to each SNP location. **Figure S8.** Average linkage disequilibrium as r-squared for each region. SNPs were assigned to gene locations. **Figure S9.** LD, measured as r^2^, plotted against physical distance. (PDF 1286 kb)

Additional file 2: Table S1. Summary of SNPs used in study of SNP annotation to imputation outcome. (XLS 26 kb)

## Abbreviations

1000G: the 1000 Genomes project
HMII: the International HapMap Project
ID: Indonesia
LD: linkage disequilibrium
MY: Malaysia
PanSNPdb: Pan-Asian SNP genotyping database
PI: The Philippines
QC: Quality control
SG: Singapore
TH: Thailand
