# Supplementary material for: Validation of genotype imputation in Southeast Asian populations and the effect of single nucleotide polymorphism annotation on imputation outcome
Source: BMC Med Genet. 2018 Feb 13;19:23. doi: 10.1186/s12881-018-0534-8 (PMC5812212; doi:10.1186/s12881-018-0534-8)
Supplement: Supplementary file 1 — Long distance LD pr. Mb separated by the chromosome for each population LD for each SNP pair with a distance between 10 kb and 1 Mb taken into account. Figure S2. Accuracy and yield of imputation between each location of SNPs. Figure S3. Comparing minor allele frequencies of SNPs between imputed and actual genotypes before quality control of imputed results separately plot by each SNP location. Figure S4. Comparing allele frequencies of SNPs between imputed and actual genotypes after quality control of imputed results separately plotted by each SNP location. Figure S5. Proportion of SNPs with lower AF after imputation vs initial AF. Figure S6. Comparing p-values between cases and controls of SNPs between imputed and actual genotypes before quality control of imputed results separately plotted by each SNP location. Figure S7. Comparing p-values between cases and controls of SNPs between imputed and actual genotypes after quality control of imputed results separately plotted according to each SNP location. Figure S8. Average linkage disequilibrium as r-squared for each region. SNPs were assigned to gene locations. Figure S9. LD, measured as r2, plotted against physical distance. (PDF 1286 kb) [file 12881_2018_534_MOESM1_ESM.pdf]

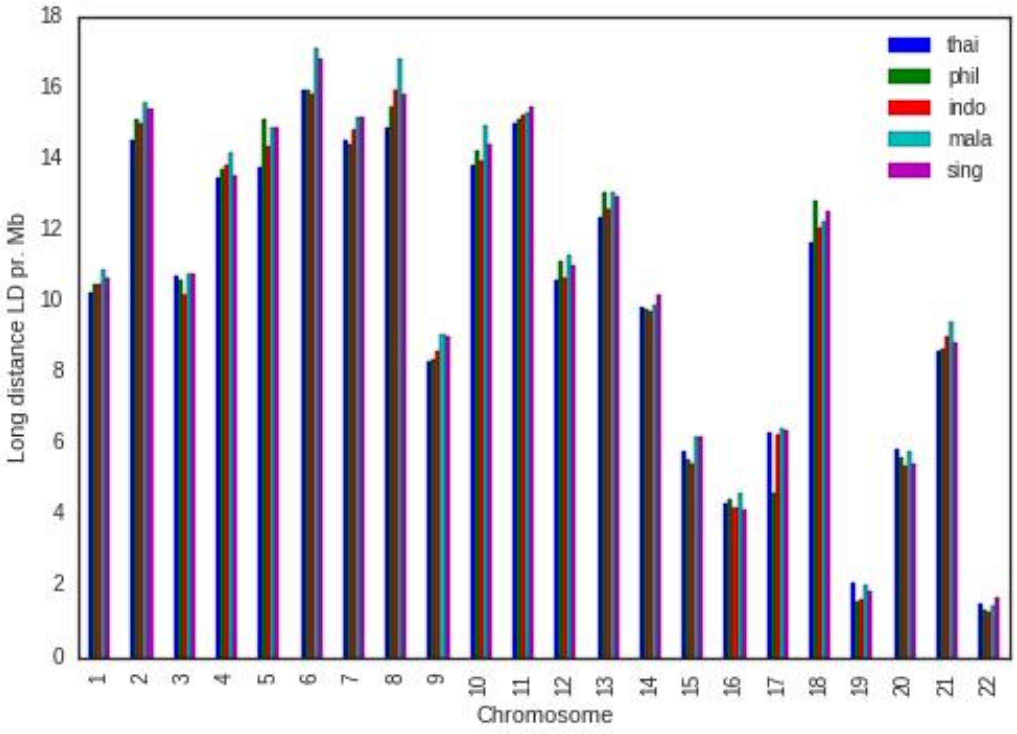

Fig. S1. **Long distance LD pr. Mb separated by the chromosome for each population LD for each SNP pair with a distance between 10 kb and 1 Mb taken into account.** The number of these pairs that had an LD > 0.2 were recorded for each chromosome and separately plotted by population for Southeast Asian populations from PanSNPdb.

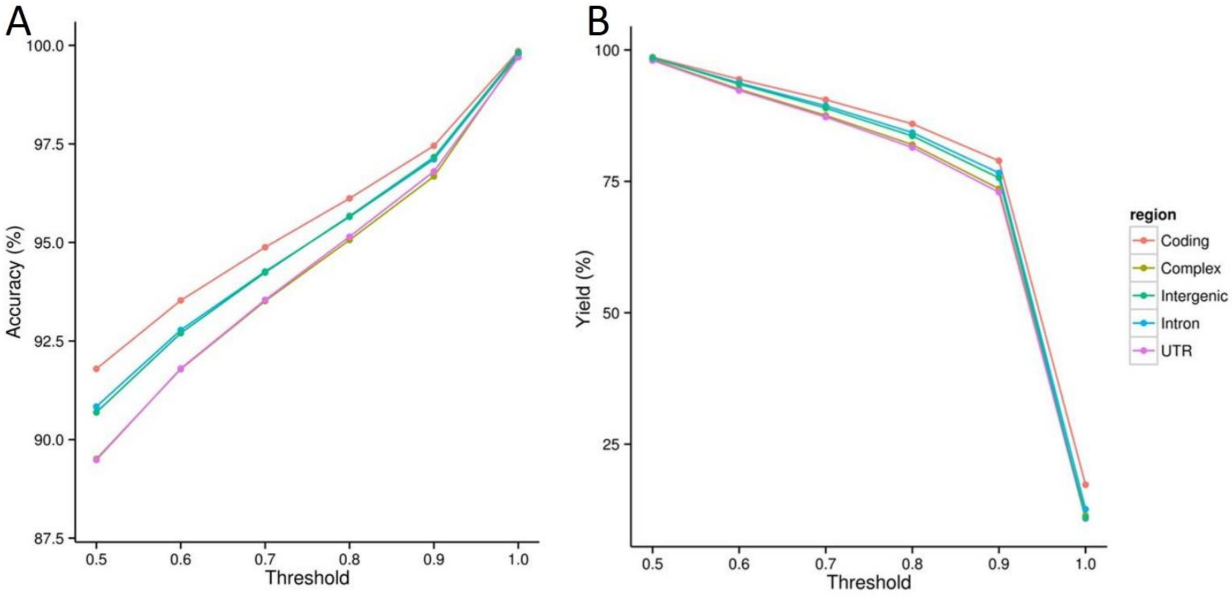

Fig. S2. Accuracy and yield of imputation between each location of SNPs. Cutting point thresholds were 0.5, 0.6, 0.7, 0.8, 0.9, and 1.

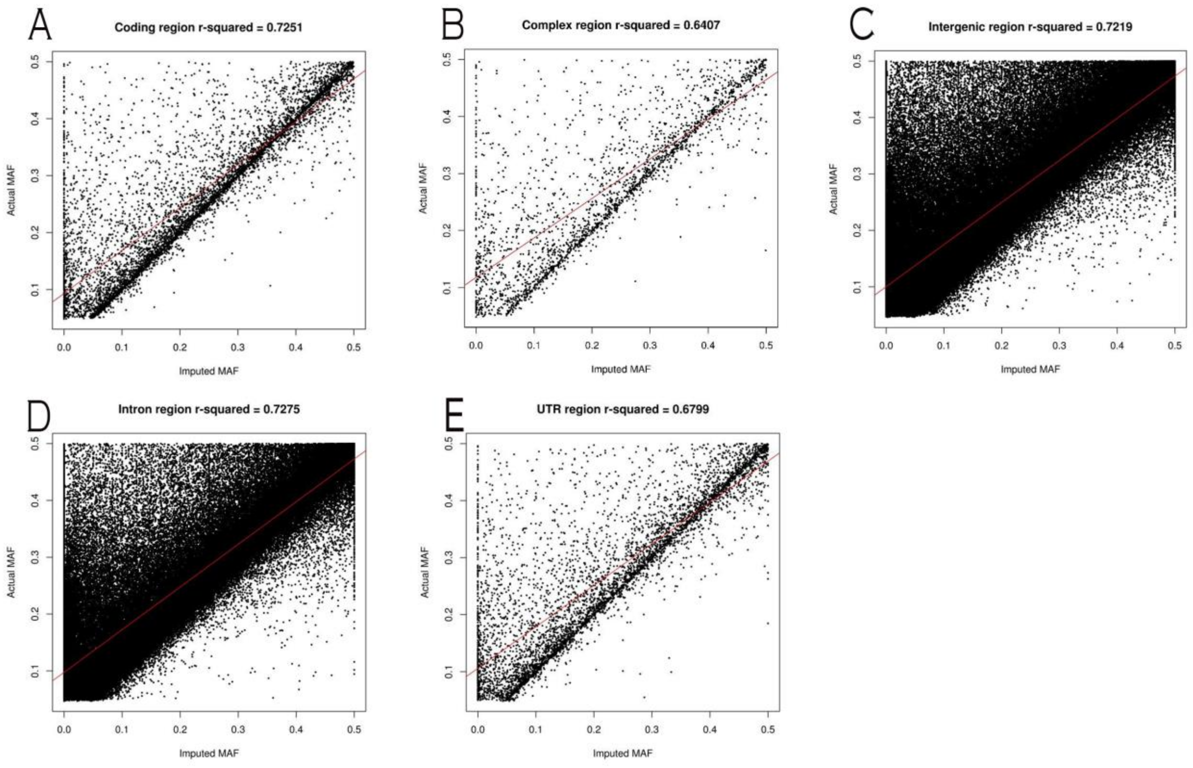

Fig. S3. Comparing minor allele frequencies of SNPs between imputed and actual genotypes before quality control of imputed results separately plot by each SNP location.

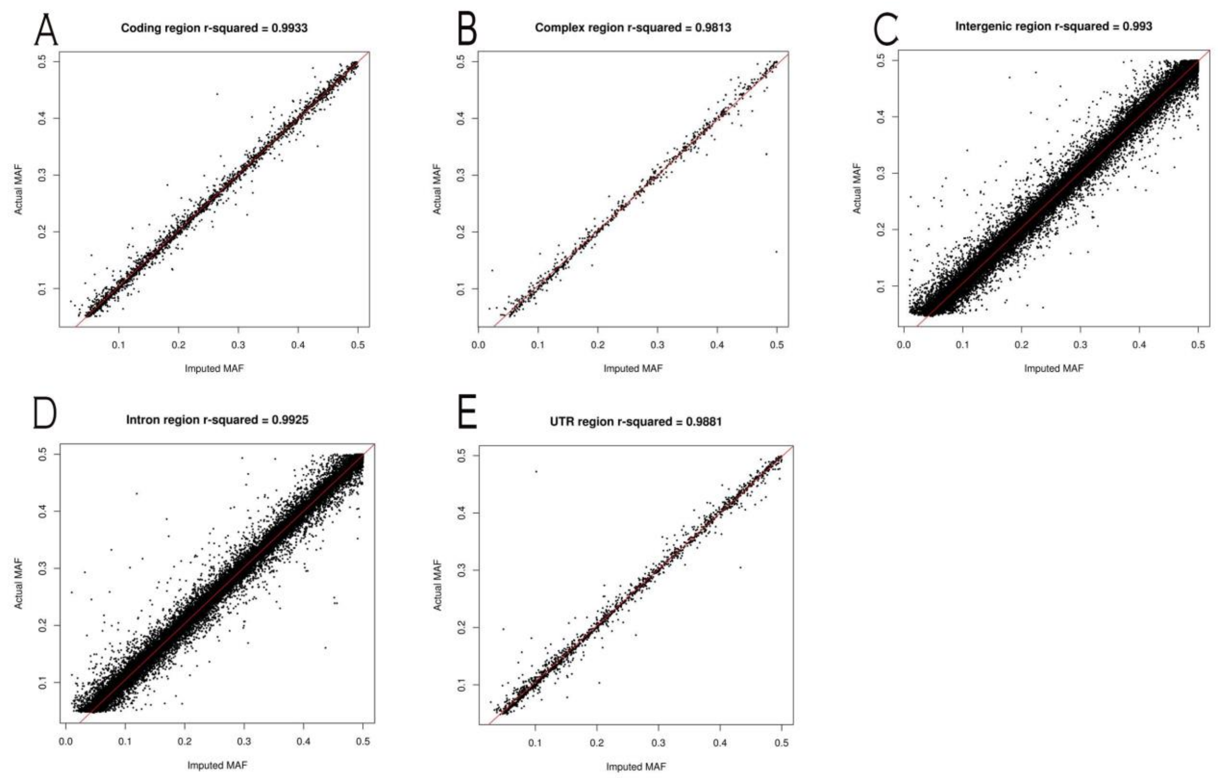

Fig. S4. Comparing allele frequencies of SNPs between imputed and actual genotypes after quality control of imputed results separately plotted by each SNP location.

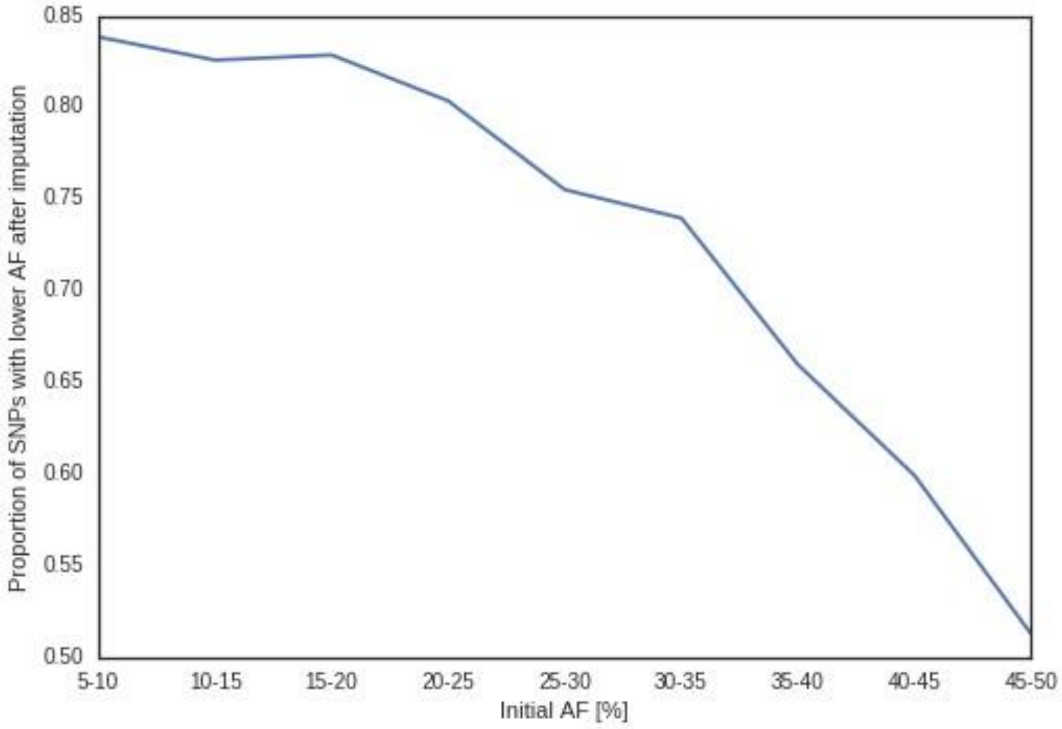

Fig. S5. Proportion of SNPs with lower AF after imputation vs initial AF.

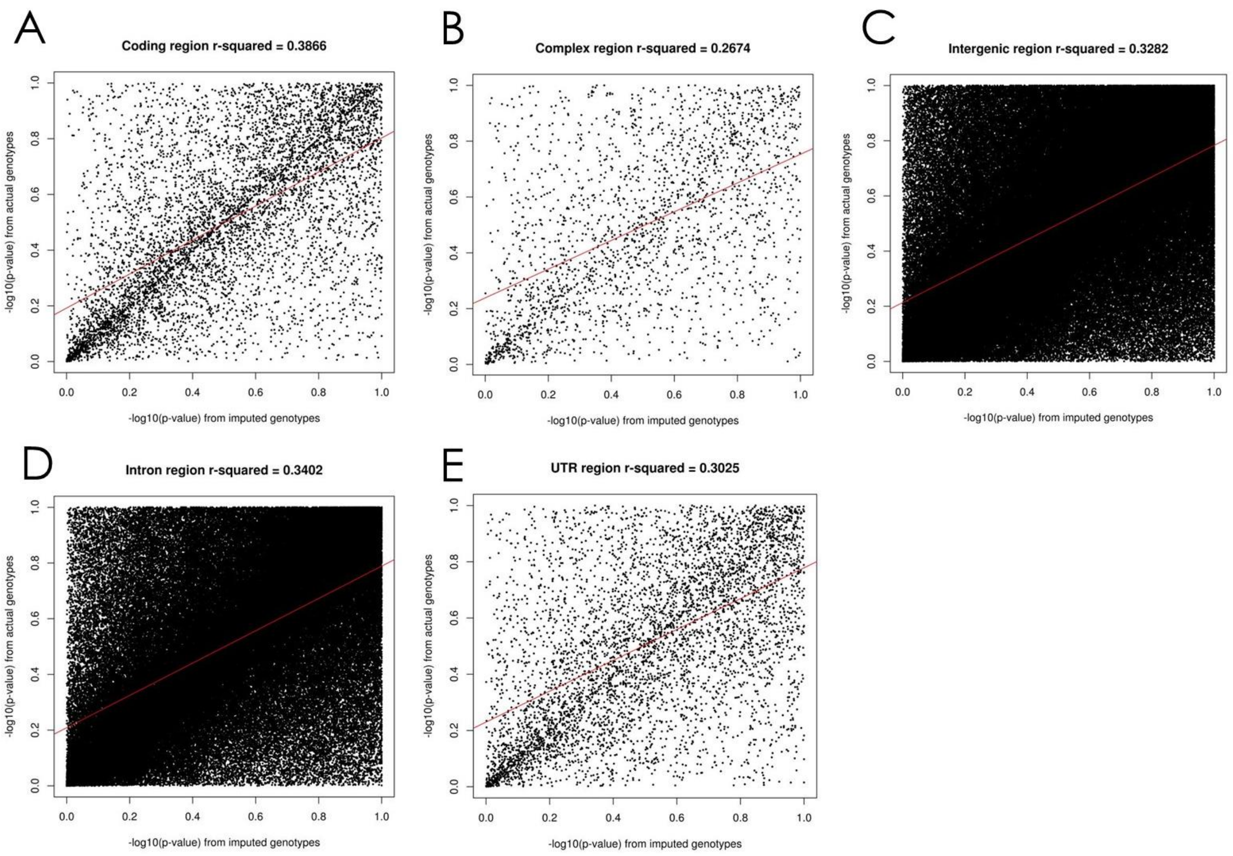

Fig. S6. Comparing p-values between cases and controls of SNPs between imputed and actual genotypes before quality control of imputed results separately plotted by each SNP location. GWAS for dengue fever vs dengue hemorrhagic fever was performed two times, including once with the original genotypes and once for the imputed datasets using the HMII reference.

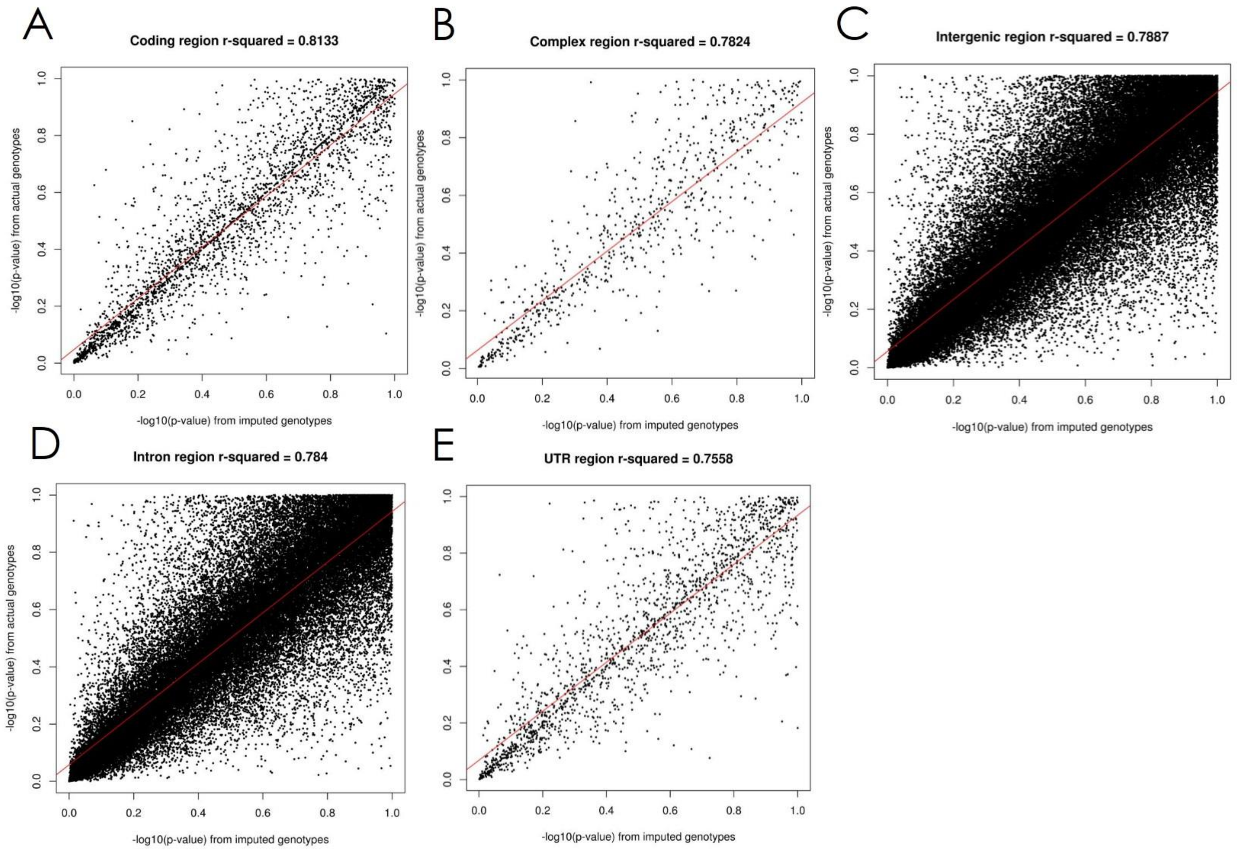

Fig. S7. Comparing p-values between cases and controls of SNPs between imputed and actual genotypes after quality control of imputed results separately plotted according to each SNP location. GWAS for dengue fever vs dengue hemorrhagic fever was performed two times, including once with the original genotypes and once for the imputed datasets using the HMII reference. The result of the imputed dataset was then performed the post-imputation quality control process.

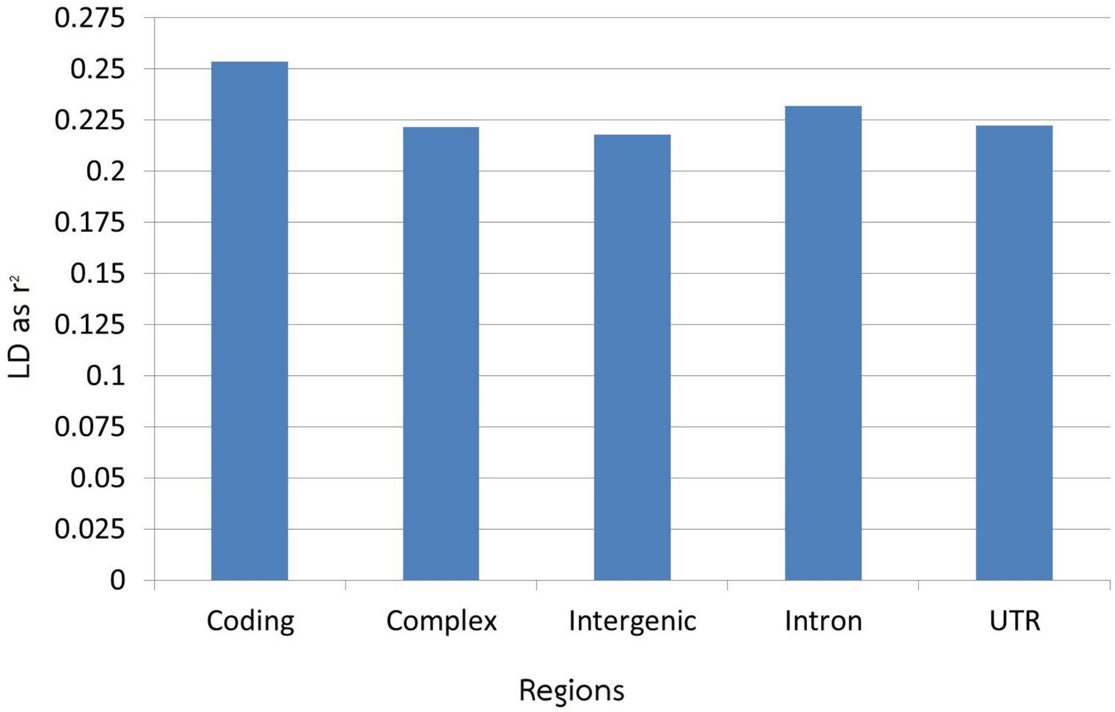

Fig. S8. Average linkage disequilibrium as r-squared for each region. SNPs were assigned to gene locations. The pairwise R-squared value was calculated for 10 neighboring SNPs in a 1 Mb window to infer the LD.

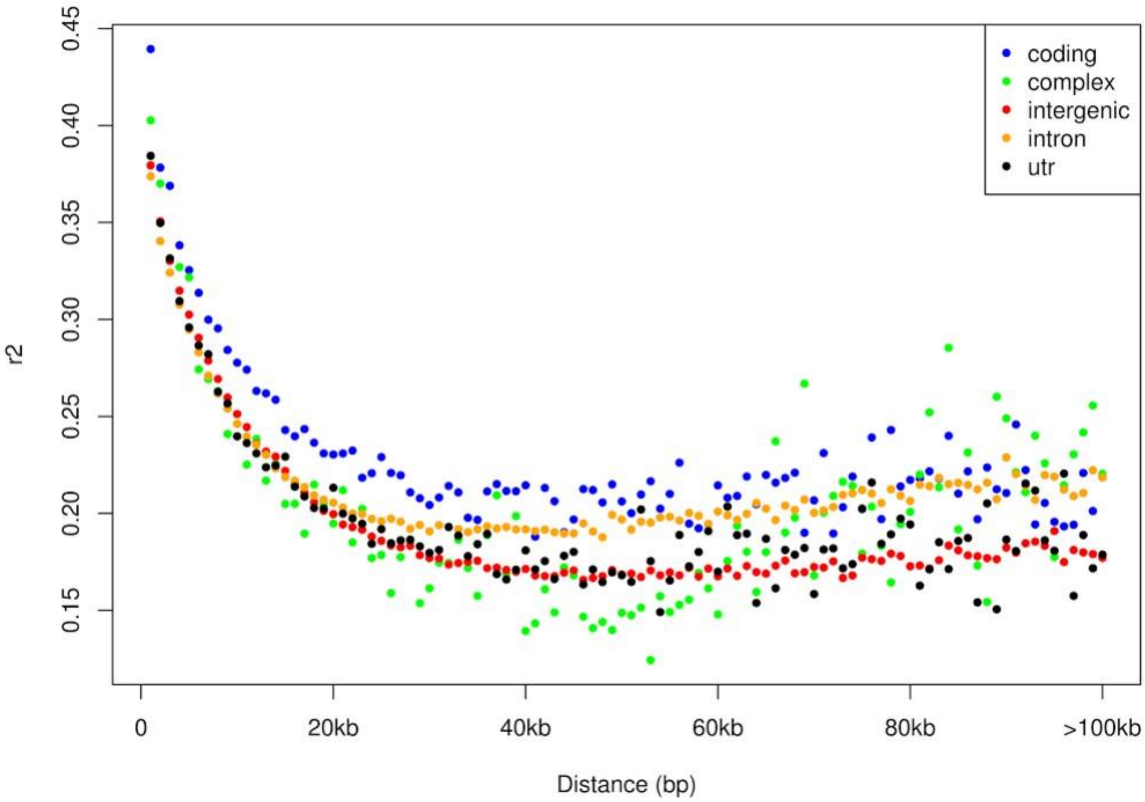

Fig. S9. LD, measured as r^2, plotted against physical distance. SNP-pairs were grouped based on their physical distance, and LD was calculated as an average per bin.
